# The Development of a Continuous Constitutive Model for Thin-Shell Components with A Sharp Change in the Property at Welded Joints

**DOI:** 10.3390/ma17081796

**Published:** 2024-04-13

**Authors:** Zhubin He, Xianggang Ruan, Jiangkai Liang, Jian Ning, Yanli Lin, Kelin Chen

**Affiliations:** State Key Laboratory of High-Performance Precision Manufacturing, School of Mechanical Engineering, Dalian University of Technology, Dalian 116024, China; ruanxg530@163.com (X.R.); 15535368969@163.com (J.L.); ningjiannj@126.com (J.N.); linyanli@dlut.edu.cn (Y.L.); kchen@dlut.edu.cn (K.C.)

**Keywords:** welded tube, continuous constitutive model, digital image correlation, free hydro-bulging, FE simulation

## Abstract

Large-dimension complex integral thin-shell components are widely used in advanced transportation equipment. However, with the dimensional limitations of raw blanks and the manufacturing process, there are inhomogeneous geometric and mechanical properties at welded joints after welding, which have a significant effect on the subsequent forming process. Therefore, in this paper, the microstructure of welded joints with a sharp property change was accurately characterized by the proposed isothermal treatment method using the BR1500HS welded tube as an example. In addition, an accurate constitutive model of welded tubes was established to predict the deformation behavior. Firstly, the heat-treated specimens were subjected to uniaxial tensile tests and the stress–strain curves under different heat treatment conditions were obtained. Then, the continuous change in flow stress in the direction of the base metal zone, the heat-affected zone and the weld zone was described by the relationship between the microhardness, flow stress and center angle of the welded tube. Using such a method, a continuous constitutive model of welded tubes has been established. Finally, the constitutive model was compiled into finite-element software as a user material subroutine (VUHARD). The reliability of the established constitutive model was verified by simulating the free hydro-bulging process of welded tubes. The results indicated that the continuous constitutive model can well describe the deformation response during the free hydro-bulging process, and accurately predicted the equivalent strain distribution and thickness thinning rate. This study provides guidance in accurately predicting the plastic deformation behavior of welded tubes and its application in practice in hydroforming industries.

## 1. Introduction

With the increasing demand for advanced transportation equipment with high reliability and long lifespan, the use of advanced forming technology to obtain complex integral thin-shell components using thin sheets and tubes as blanks is developing rapidly [1,2,3]. As a typical raw blank, thin-walled welded tubes are increasingly being applied in aviation (the intake and exhaust systems of advanced fighters), aerospace (lunar rover torus receptacle) and automotive industries (automotive sub-frame) due to their attractive benefits of low production costs, high manufacturing efficiency and reliable quality [4,5]. Welded tubes can be manufactured via various welding processes, including laser welding, arc welding and resistance welding [6,7,8]. The microstructure and mechanical properties of the weld zone and the heat-affected zone (HAZ) are significantly influenced by thermal cycling during welding [9]. Numerous studies have confirmed the existence of notable distinction within the microstructure, microhardness, tensile strength and elongation of the weld zone, the HAZ, and the base metal zone [10,11]. It has been demonstrated that the inhomogeneous characteristics undergo continuous change in these specific zones.

Nowadays, the effect of inhomogeneous mechanical properties on the deformation behavior of welded tubes has been extensively studied, and finite-element (FE) simulation has become one of the most commonly used methods [12]. The reliability of the results of a numerical simulation depends on the accuracy of the adopted constitutive model, and accurate and sufficient experimental data are required to establish an accurate constitutive model [13,14]. Establishing an accurate and effective constitutive model for welded tubes that can simultaneously account for the continuous material property change over the base metal zone, the HAZ and the weld zone has become an important issue at present. The main difficulty in this process is to simulate the weld zone and the HAZ as accurately as possible, both in need to improve the calculational accuracy and to ensure computational efficiency [15].

A variety of test methods for determining weld zone and HAZ properties have been proposed in the published literature on FE analysis of weld metals. In the first method, the sub-sized specimen tensile tests were used to determine the properties of weld alone, HAZ alone and mixed tensile specimens [16]. For instance, Wei et al. [17] determined the properties of the weld zone by performing tensile tests on weld alone specimens cut on the steel sheet. However, due to the constraints of an irregular and narrow weld zone cross-section, it was often not possible to cut a tensile specimen containing only the weld. In addition, the weld zone mechanical properties were influenced by the width of the specimen, which led to differences in the results for specimens of different widths [18]. The second method was based on microhardness test and called the empirical formula, which was proposed by Reis et al. [19]. Using this method, Khalfallah [20] determined the weld mechanical properties of S235JR low-carbon steel tubes. The microhardness test method based on empirical relationships has the benefits of simplicity and convenience. In addition, Tuninetti et al. [21,22] highlighted that it was possible to acquire the flow stress and elastic modulus from the material hardness value obtained by nanoindentation testing. The third method was the rule of mixtures proposed by Abdullah et al. [23], which assumed the iso-strain in the perpendicular tensile direction for mixed tensile specimens. Nevertheless, the method assumes that the HAZ or base metal was the material neighboring the weld. Therefore, this assumption resulted in the weld’s properties being sensitive to both the weld zone size and the specimen dimensions, which influenced the test accuracy.

In recent years, with the development of property test methods and the wide application of welded tubes, the research on the inhomogeneous properties of welded tube joints has received extensive attention. Kim et al. [24] expanded upon the utilization of the rule of mixtures to evaluate the HAZ properties in welded tubes based on mixed specimen tensile tests. Subsequently, Zhan et al. [25] improved the rule of mixtures by dividing the HAZ into multiple zones to achieve the mechanical properties change within the HAZ of the welded tube. Using the above-modified manner, the plastic constitutive relationship of the Q215 welded tube was determined. The results demonstrated that the method could accurately reveal the change in flow stress over the HAZ and reduced the influence of the mixed specimen width on the flow stress–strain curve to a certain extent. Ren et al. [26] used the improved rule of mixtures, numerical control rotary draw bending tests and FE simulations to investigate the constraint effect of the weld and the HAZ on the bending property of the welded tube QSTE340.

For accurate characterization of mechanical properties of the base metal zone, the HAZ and the weld zone of the welded tube, it was essential to consider the change in material properties in the HAZ and its continuity. Therefore, a continuous and accurate constitutive model of the welded tube within its cross-section zone was needed. Xing et al. [27] established a continuous constitutive model for welded tubes according to the rule of mixtures and the integral principle, combined with the continuous distribution of the microhardness of the welded joint. The constitutive model was evaluated by bending deformation tests on the QSTE340 welded tube. The comparison of the simulation and experimental results shows that the continuous constitutive model had a higher prediction accuracy. Liu et al. [28] established the continuous constitutive relationship of rectangular welded tubes of QSTE700 high-strength steel by the rule of mixtures and nanoindentation test. Subsequently, the rotary draw bending process and nanoindentation test verified the reliability of the established continuous constitutive model. Under the above analysis, it could be seen that the characterization for the base metal zone, HAZ and weld zone properties in the welded tube was achieved via combining with the rule of mixtures, and established the continuous constitutive model of the welded tube. However, the accuracy of determining welded joint properties based on the rule of mixtures depended on the accuracy of measuring the area of each zone and was sensitive to the size of the mixed specimen [29]. Therefore, an accurate method to determine the mechanical properties of welded tube joints is needed to establish the welded tube continuous constitutive model.

To address the current limitations of commonly used discrete constitutive models in welded tubes, an isothermal treatment method was proposed to characterize the microstructure of welded tube joints with a sharp property change in BR1500HS laser-welded tubes directly. By using the relationship between the standard tensile specimen’s tensile properties (strength coefficient *K* and hardening index *n*), microhardness and welded tube center angle, in combination with the microstructure of the welded joints, the continuous constitutive model of the BR1500HS laser-welded tubes was determined. The established constitutive model was compiled as a user material subroutine into FE simulation, and the equivalent strain and wall thinning rate during the tube free hydro-bulging process of welded tubes was analyzed. The reliability of the established continuous constitutive model was also validated. This study will provide guidance in accurately predicting the plastic deformation behavior of welded tubes and its application in practice in hydroforming industries.

## 2. Materials and Methods

### 2.1. Materials

In this study, the commercial boron steel BR1500HS uncoated sheet with a thickness of 3.2 mm manufactured by Baoshan Iron and Steel Co., Ltd. (Shanghai, China) was used. Table 1 lists the chemical composition of the BR1500HS sheet. The original tube with an initial outer diameter of 90 mm was prepared using the U-O method [30,31,32] and laser welding method.

### 2.2. The Isothermal Treatment Method

During the straight seam welding process, the molten pool metal cools rapidly along the direction perpendicular to the welding direction. The rapidly cooled weld zone after laser welding of boron steels is mainly martensite [33,34,35]. However, the microstructure of the HAZ varies with the distance from the welding heat source. The HAZ zone metal with a nearer distance from the welding heat source will be fully austenitized during the welding when the peak heating temperature exceeds *Ac*_3_. Thus, the metal in this zone will transform into martensite during rapid cooling. For HAZ metals far from the welding heat source, the peak heating temperatures range from *Ac*_1_ to *Ac*_3_, and only part of the original microstructure undergoes austenitization during welding. Therefore, the microstructure consists of ferrite–martensite mixtures during subsequent cooling [36,37].

**Figure 1:**
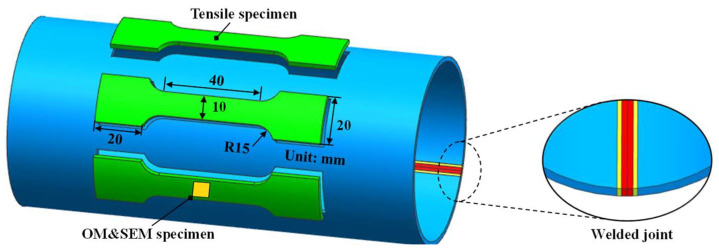
Positions and dimensions of uniaxial tensile specimens.

In this paper, the specimens of single-phase martensite and ferrite–martensite mixture microstructure in welded joints were obtained by the isothermal treatment method. The volume fraction of each phase of the ferrite–martensite mixture microstructure varied by controlling the ferrite transformation time. Therefore, the microstructure of the HAZ with a sharp property change could be effectively characterized. Figure 1 shows the positions and dimensions of the arc-shaped tensile specimens cut along the longitudinal direction of the original tube for isothermal treatment.

**Figure 2:**
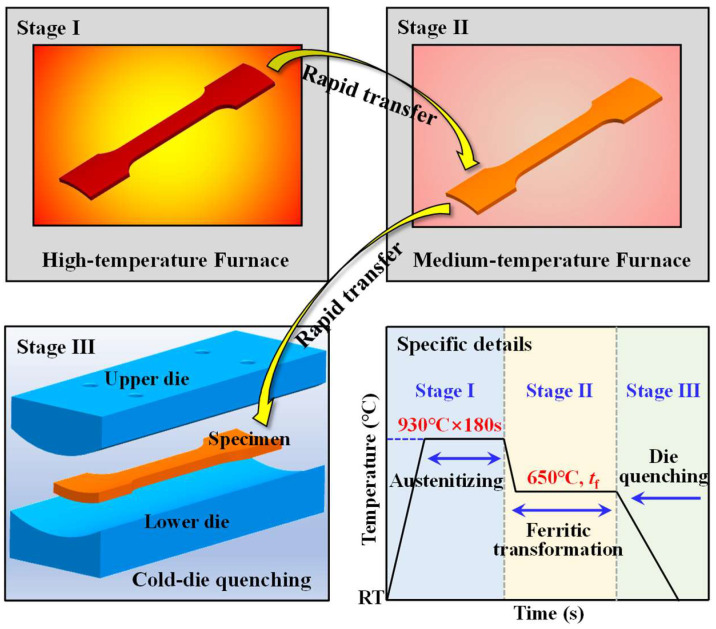
Schematic of the isothermal treatment cycle.

The isothermal treatment process is schematically shown in Figure 2, and different volume fractions of ferrite–martensite mixture microstructure could be obtained at different holding times *t*_f_. Two heating furnaces and an arc-shaped surface cold quenching die were used for isothermal treatment of the specimens. The heat treatment specimen preparation process involves three stages: (I) Heating the arc-shaped specimen of the original tube to 930 °C in a high-temperature furnace and holding it for 3 min to achieve complete austenitization; (II) Transferring the fully austenitized specimen rapidly to a medium-temperature furnace (constant 650 °C) for holding (0 s, 30 s, 60 s, 90 s) to achieve the ferrite microstructure transformation; (III) Removing the specimen quickly and transferring it to an arc-shaped cold quenching die. The specimen was cooled under a contact pressure of 15 MPa between the cold die with a holding time of 15 s to achieve the martensite transformation. To prevent warming of the cold quenching die due to continuous heating during successive experiments, the die was cooled using room temperature high-pressure gas between the experiment intervals.

### 2.3. Microstructural and Mechanical Characterization

To establish an accurate continuous constitutive model of welded tubes, the mechanical properties and microstructure of welded tube joints need to be accurately characterized. Specimens for metallographic and microstructural analysis were prepared using standard mechanical polishing procedures. The specimens were first ground using SiC papers (400–2000 grit size) and then polished with 0.5 μm diamond abrasive paste. Subsequently, specimens for microstructure observation were etched using a 4% volume fraction of nitric acid alcohol solution for 15 s. The microstructure of the specimens was characterized using Lecia optical microscope (OM) (Leica Instruments Co., Ltd., Munich, Germany) and the JSM-7900F scanning electron microscope (SEM) (Nippon Electronics Corporation, Tokyo, Japan). To enhance the measurement accuracy and reflection of the microhardness distribution in the base metal zone, the HAZ and the weld zone, the specimen with an arc-shaped section was used. According to GB/T4340.1-2009 [38], the Vickers hardness test was carried out using an HVS-1000 microhardness tester (Laizhou (Shandong) Metalreader Testing Instrument Co., Ltd., Shandong, China) on the arc-shaped section specimens under 200 g load and 10 s holding time. Different measurement intervals were adopted in different zones of the welded joint, where the weld zone and the HAZ were measured at an interval of 0.05 mm and the base metal zone with an interval of 0.1 mm. The uniaxial tensile specimen was processed according to the standard GB/T 228.1-2021 [39], with the dimensions of 20 mm (gauge length) × 10 mm (width) × 3.2 mm (thickness), as shown in Figure 1. Room temperature axial tensile tests were conducted on heat-treated specimens and original base metal specimens using the LE5105 material tension tester (Lishi (Shanghai) Scientific Instrument Co., Ltd., Shanghai, China) equipped with a digital image correlation (DIC) strain measurement system to record the strain field during tensile tests. The DIC strain measurement system with a resolution of the images to 40 μm/pixel and is close to the literature [40]. The crosshead speed of the material tension tester was set to 2 mm/min.

The tube hydro-bulging test has the same boundary conditions as the hydroforming process in practice, thus the deformation behavior of the tube can be evaluated by this test. The ends of the tube were sealed with two rigid conical punches, and the tube was freely bulged under the internal pressure medium. The axial sealing force and internal pressure of the tube were measured by a load sensor and pressure sensor, respectively with maximum values of 500 kN and 160 MPa. In addition, to accurately measure the strain change during the free bulging deformation of the tube, the DIC strain measurement system was used to analyze the full-field strain distribution. Random speckles were sprayed on the tube with black paint before bulging and a white undercoating was painted to enhance the image contrast. With two CCD cameras, the displacement and strain of the tube can be calculated by comparing speckle images before and after bulging.

### 2.4. Describing the Continuous Change in Flow Stress

**Figure 3:**
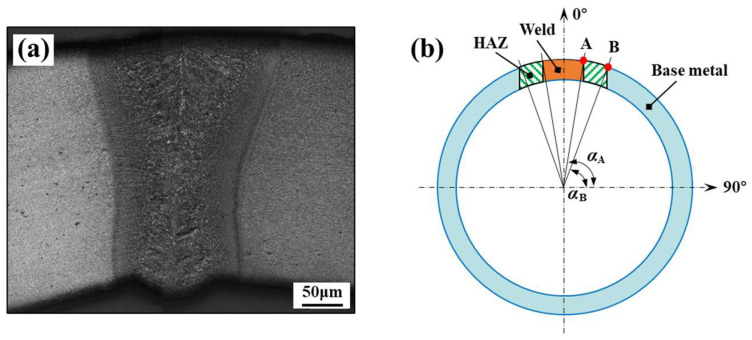
Cross-section of the welded tube: (**a**) etched metallographic section of welded joint, (**b**) partition schematic diagram of welded joint.

As mentioned earlier, there is a sharp property change within the welded joint zone. The shapes of the weld zone and the HAZ in welded tubes are close to arc-shaped (see Figure 3a). In this study, the continuous change in spatial coordinates on the welded tube was used for describing the continuous change in flow stress, as shown in Figure 3b. Assuming that the coordinates of points A and B on the welded tube were (*x*_1_,*y*_1_) and (*x*_2_,*y*_2_), respectively, the corresponding angles of points A and B were denoted as Equations (1) and (2), respectively:

(1) $$\alpha_{A}=atan\left(y_{1}/x_{1}\right)$$

(2) $$\alpha_{B}=atan\left(y_{2}/x_{2}\right)$$

Different constitutive models are satisfied in different zones of the welded tube joint. Here, the *σ*_w_, *σ*_h_ and *σ*_b_ were used to represent the flow stress for the weld zone, the HAZ and the base metal zone, respectively, and it was assumed that the stress–strain relationship satisfies the Hollomon model. Considering the symmetry of the shapes and properties of the welded tube, the welded tube continuous constitutive model could be expressed as Equation (3):

(3) $$\left\{ \begin{array}{l} \sigma_{w}=K_{w}\varepsilon^{n_{w}}\;for\;(0^{{}^{\circ}}\leq \alpha <\alpha_{A})\\ \sigma_{h}=K(\alpha )\varepsilon^{n(\alpha )}\;for\;(\alpha_{A}\leq \alpha \leq \alpha_{B})\\ \sigma_{b}=K_{b}\varepsilon^{n_{b}}\;for\;(\alpha_{B}<\alpha \leq 180^{{}^{\circ}}) \end{array}\right.$$

where *K* is the strength coefficient and *n* is the hardening index.

The accurate description of the mechanical properties and their change trends in the HAZ is the key to establishing the welded tube continuous constitutive model accurately. Specimens with different proportions of ferrite–martensite mixture microstructure were obtained using the isothermal treatment method to determine the flow stress in the HAZ. In addition, it was necessary to determine the specific locations of various flow stress obtained above in the HAZ.

The relationship between microhardness and flow stress was approximately constant [41,42,43]. That is, the flow stress was in proportion to the microhardness, as shown in Equation (4). The hardness change curve between the microhardness and center angle of the welded tube from the weld center to the base metal could be obtained by the microhardness test in Section 2.3. The corresponding hardness of the HAZ could be calculated from Equation (4) if the flow stress in the HAZ has already been identified. Meanwhile, the relationship between the flow stress and the center angle of the welded tube in the HAZ was determined for different proportions of ferrite–martensite mixture microstructure.

(4) $$\sigma_{w}=\sigma_{h}\frac{HV_{w}}{HV_{h}}$$

where *HV*_w_ is the Vickers hardness in the weld zone and *HV*_h_ is the Vickers hardness in the HAZ.

## 3. Results

### 3.1. Microstructure in the Welded Joint

**Figure 4:**
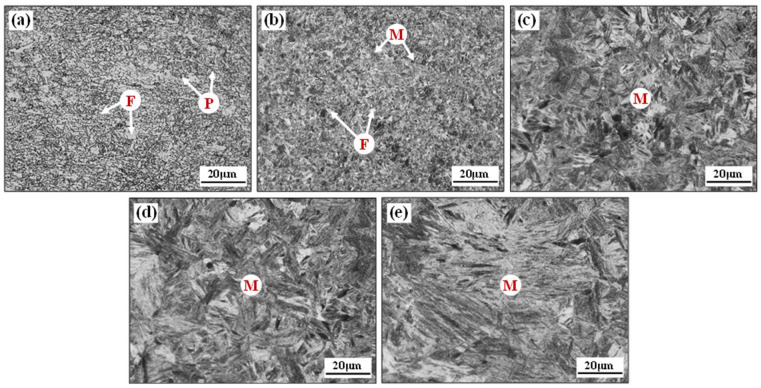
Microstructures of the base metal zone (**a**), the sub-critical HAZ (**b**), the inter-critical HAZ (**c**), the super-critical HAZ (**d**) and the weld zone (**e**).

Figure 4 shows the metallographic distribution of the laser-welded tube joint from the center of the weld to the base metal. The typically mixed microstructure is ferrite (F) and pearlite (P) in the base metal zone and slat martensite (M) in the weld zone. According to the peak heating temperature that the BR1500HS tubes underwent during welding, the HAZ was divided into three zones: the sub-critical HAZ, the inter-critical HAZ and the super-critical HAZ.

The inter-critical HAZ and the super-critical HAZ underwent a temperature exceeding *Ac*_3_ during welding thermal cycling, and the nucleated austenitic microstructure transformed into martensite in subsequent cooling. In particular, the super-critical HAZ approach to the fusion line, which undergoes a peak heating temperature between the superheat temperature and the melting point, thus leads to severe growth of austenite grain. However, the inter-critical HAZ undergoes a peak heating temperature between *Ac*_3_ and superheat temperature during the welding thermal cycle, resulting in a finer martensitic microstructure. In the sub-critical HAZ, the original ferrite was affected to different degrees and the microstructure mainly consisted of ferrite–martensite mixture microstructure. There was a significant sharp change in the mechanical properties within this extremely narrow zone. Accurately characterizing the microstructure and mechanical properties in this zone is crucial for establishing an accurate constitutive model of welded tubes.

**Figure 5:**
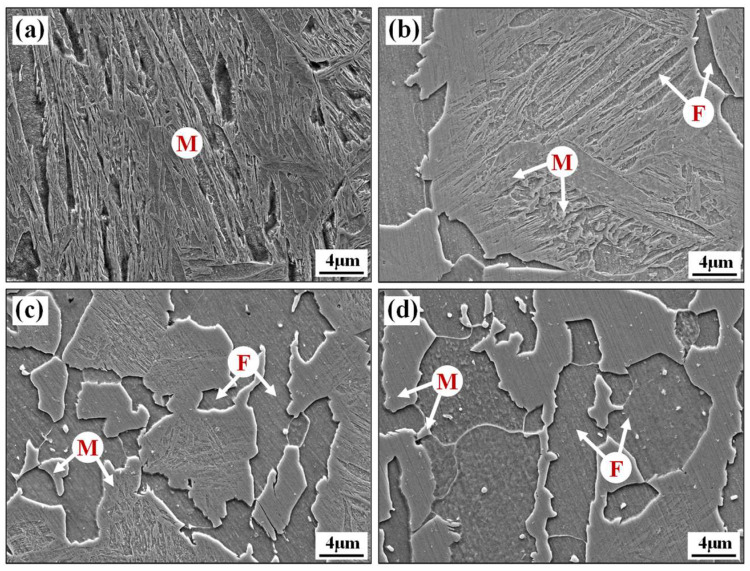
Ferritic-martensitic microstructure of specimens with different ferritic transformation time: (**a**) 0 s, (**b**) 30 s, (**c**) 60 s, and (**d**) 90 s (area size 40 × 30 μm at a magnification of 3000×).

The SEM microstructure of the weld zone and sub-critical HAZ specimens obtained by isothermal treatment is shown in Figure 5. The isothermal transformation time of 0 s corresponds to the microstructure of the weld zone, which exhibits a typical microstructure of slat martensite for laser welding. The isothermal transformation times of 30 s, 60 s and 90 s correspond to the ferrite–martensite microstructure of various positions of the sub-critical HAZ, respectively. The volume fractions of the ferrite and martensite phases under different isothermal transformation time conditions were determined by processing the SEM data using ImageJ 1.32i quantitative image analysis software [44,45]. The brighter zones in Figure 5b–d are the martensitic phase and the darker zones correspond to the ferrite phase, and the exact phase volume fraction is shown in Table 2.

### 3.2. Microhardness Distribution in the Welded Joint

**Figure 6:**
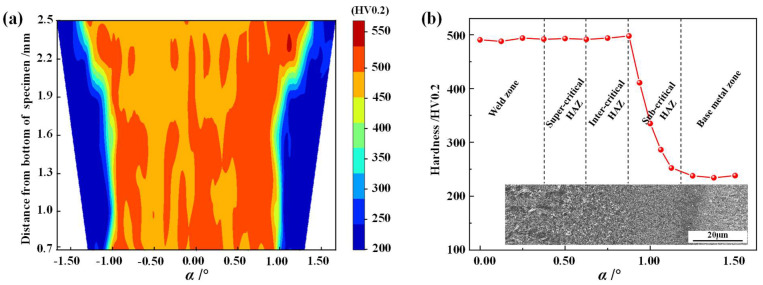
Hardness distribution: (**a**) hardness map; (**b**) hardness distribution and microstructure morphology.

To determine the width of each zone of the welded joint, the Vickers hardness test was carried out on welded tube arc-shaped section specimens according to Section 2.3. Figure 6a shows the hardness map of the BR1500HS laser-welded tube joint, which was stable at approximately 500 HV0.2 over a wide range (± 1°) from the center of the weld. In addition, the hardness of the specimen decreased rapidly to about 230 HV0.2 along the width direction in a very narrow zone.

To observe the hardness distribution from the weld center to the base metal more clearly, the hardness data from the weld to the base metal at the lowest position (0.7 mm from the bottom of the specimen) in the hardness map of Figure 6a was analyzed. Since the symmetrical distribution of hardness on both sides of the BR1500HS welded tube, only the hardness distribution of the half tube was analyzed, as shown in Figure 6b. In addition, the hardness distribution of the welded tube was analyzed in combination with the microstructure morphology of the corresponding locations. It was shown that the hardness of the weld zone, the super-critical HAZ and the inter-critical HAZ was significantly higher than that of the base metal zone. After welding, the microstructure of the above three zones with a width of approximately 0.7 mm (0.88°) consists mainly of martensite, which leads to significantly greater hardness in these zones than the base metal zone with a mixture of ferrite and pearlite. In addition, the hardness increased slightly from the super-critical HAZ to the inter-critical HAZ. This is mainly because the grain size of the inter-critical HAZ is smaller than that of the super-critical HAZ due to different peak temperatures of the welding thermal cycle in the two zones [36]. According to the Hall-Petch equation, as described by Hansen [46], the smaller the average value of the grains, the higher the strength of the material. Therefore, the inter-critical HAZ has a higher hardness compared to the super-critical HAZ and the weld zone. In particular, in the sub-critical HAZ with a width of about 0.4 mm (0.5°), the hardness decreases significantly, with a difference of 2.12 times between the maximum and minimum hardness values.

### 3.3. The Establishment of Continuous Constitutive Model of Welded Tubes

**Figure 7:**
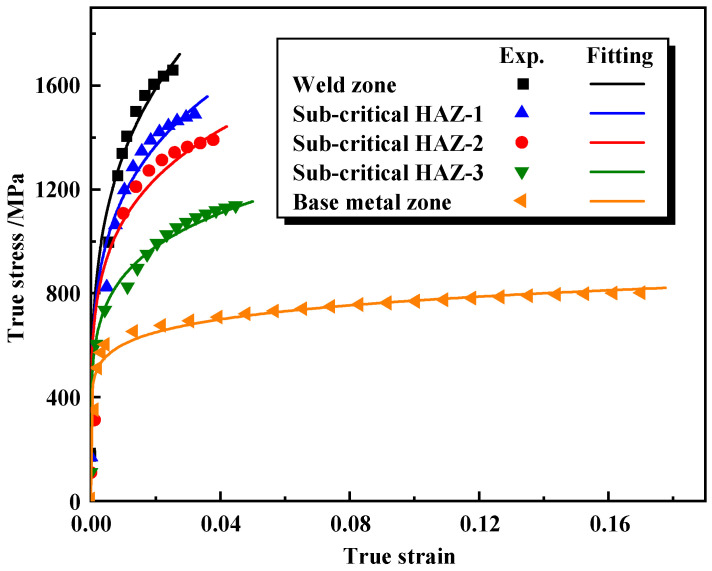
The true stress–strain curves of the BR1500HS welded tube.

Combined with the microstructure of the welded tube joint in Figure 4 and the hardness distribution in Figure 6b, the weld zone, the super-critical HAZ, and the inter-critical HAZ were unified as the weld zone. The stress–strain relationship in the weld zone, the sub-critical HAZ and the base metal zone tested by uniaxial tensile testing are shown in Figure 7. By fitting the true stress–strain curves of the specimens in the weld zone, the sub-critical HAZ and the base metal zone, it was found that Hollomon’s Equation (5) describes the change and extrapolation trend very well. As shown in Table 3, the fitted results for each zone of the welded tube indicate that, the *K* and *n* decrease from the weld to the base metal gradually.

(5) $$\sigma =K\varepsilon^{n}$$

**Figure 8:**
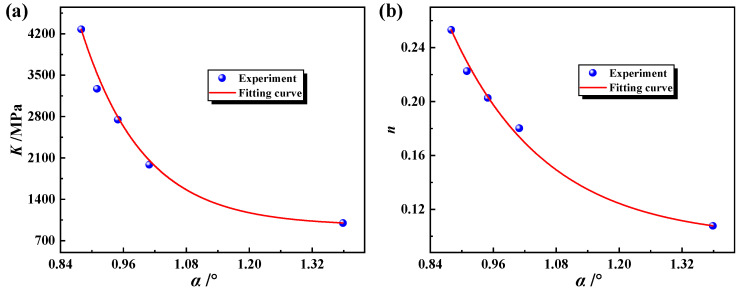
Distribution of plastic parameters of the welded tube and corresponding fitted curves: (**a**) the change of *K* from the weld zone to the base metal zone, (**b**) the change of *n* from the weld zone to the base metal zone.

To establish a constitutive model that accurately describes the continuous change in flow stress in the weld zone, the HAZ and the base metal zone, the change in *K* (see Figure 8a) and *n* (see Figure 8b) from the weld zone to the base metal zone in Table 3 was fitted using Origin software 2021, as shown in Figure 8. Several decay functions can be used to describe the change in *K* and *n* along the weld to the base metal. For convenience, two typical decay functions-exponential functions were used to describe this changing law, as shown in Equations (6) and (7).

(6) $$K=938.986+5611805.656e^{-8.441\alpha }$$

(7) $$n=0.097+19.817e^{\left(-\alpha /0.182\right)}$$

where *α* is the angle distance from the center of the weld, ranging from 0.88° to 1.38°.

According to the proposed method above for establishing the welded tube continuous constitutive model, combined with the fitting results of plasticity parameters, the continuous constitutive model of BR1500HS welded tube was obtained with the weld as the symmetry center, as shown in Equation (8).

(8) $$\left\{ \begin{array}{l} \sigma_{w}=4273.656\varepsilon^{0.253}\;for\;(0{}^{\circ}\leq \alpha \leq 0.88{}^{\circ})\\ \sigma_{h}=(938.986+5611805.656e^{-8.441\alpha })\varepsilon^{0.097+19.817*e^{\left(-\frac{\alpha }{0.182}\right)}}\;for\;(0.88{}^{\circ}\leq \alpha \leq 1.38{}^{\circ})\\ \sigma_{b}=987.972\varepsilon^{0.107}\;for\;(1.38{}^{\circ}\leq \alpha \leq 180{}^{\circ}) \end{array}\right.$$

**Figure 9:**
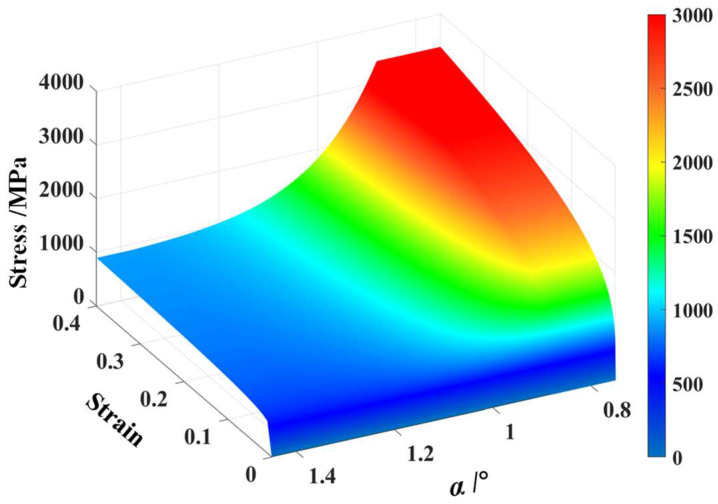
The stress–strain curve along the welded tube center angle.

Depending on Equation (8), the distribution of stress–strain curves along the BR1500HS welded tube center angle was determined, as shown in Figure 9. It can be seen in Figure 9, the flow stress in the weld is the maximum at the same strain, and the flow stress varies continuously and decreases from the weld to the base metal gradually.

## 4. Discussion

### 4.1. Establishing the Experiment and the FE Model

To evaluate the accuracy of the established continuous constitutive model for BR1500HS welded tube, the model was applied to the FE simulation of the free hydro-bulging process of the welded tube. By evaluating the accuracy of predicting the deformation of tube free hydro-bulging experiments with discrete and continuous models, the benefits of continuous constitutive model in simulating continuous inhomogeneous materials were demonstrated.

#### 4.1.1. Tube Free Hydro-Bulging Tests

**Figure 10:**
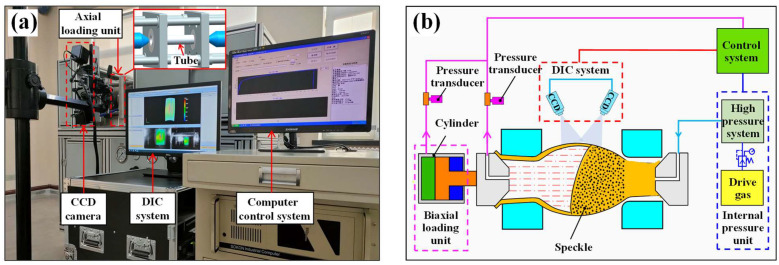
Testing system for tube free hydro-bulging with fixed-ends: (**a**) test device and (**b**) schematic diagram.

To determine the deformation characteristics of the welded tube during the bulging process, the hydro-bulging test of BR1500HS welded tube with different length-diameter ratios was carried out on the self-designed tube hydro-bulging test system. The tube free hydro-bulging with fixed-ends was a biaxial stress state. The hydro-bulging test system utilized in this study is depicted in Figure 10 and mainly consists of the axial loading unit, high pressure system, DIC strain measurement system and control system. The control system combines an axial loading device with a high pressure system to achieve precise control of the stress path.

#### 4.1.2. The FE Model

**Figure 11:**
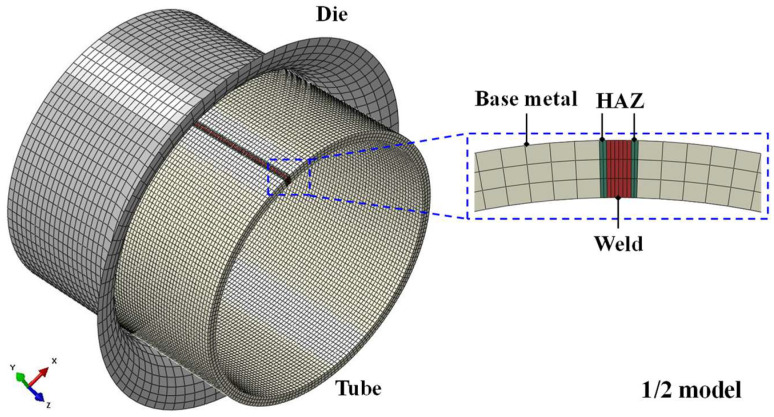
The FE model for free hydro-bulging of BR1500HS welded tube.

To analyze the reliability of the established welded tube continuous constitutive model, the model was compiled into the FE simulation to simulate the tube free hydro-bulging process. Meanwhile, a discrete FE model of the welded tube constitutive model was established, in which the HAZ was considered a homogeneous material and the flow stress was averaged over the weld and the base metal. As Figure 11 shows, a 3D FE model was built on the ABAQUS/Explicit platform, which included the end restraint die and the welded tube. The initial diameter *D*_0_ and the initial thickness *t*_0_ of the welded tube are 90 mm and 3.2 mm, respectively. The length of the bulging zone is defined as *L*. The length-diameter ratio *λ* of the validated model in this section is 1 (*λ* = *L*/*D*_0_). Considering the geometric symmetry, only 1/2 of the welded tube free hydro-bulging FE model was simulated. In balancing the simulation accuracy and computational efficiency, the element sizes of the weld zone, the HAZ and the base metal zone of the welded tube were meshed to 0.1 mm, 0.1 mm and 0.7 mm, respectively. The tube was divided into three layers in the thickness direction with a total of 94,500 elements. In the simulation, the die was defined as a discrete rigid body meshed with 4-node 3D bilinear rigid quadrilateral element of type R3D4. The laser-welded tube was defined as a deformable body meshed with a solid element of type C3D8R of reduced 8-node linear with hourglass control.

**Figure 12:**
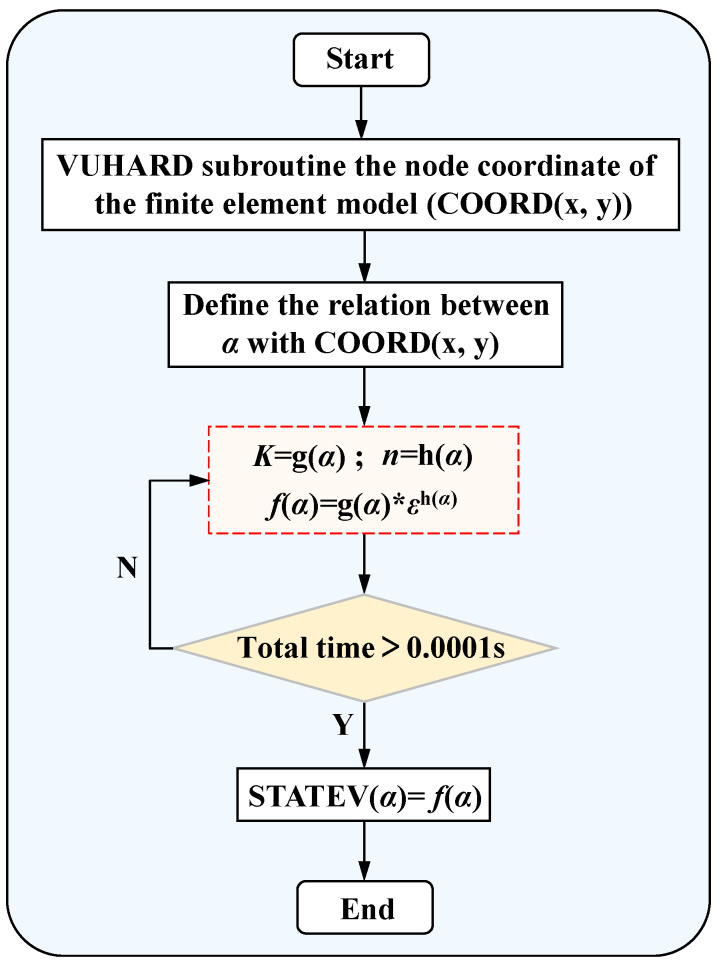
The flowchart of the VUHARD subroutine.

In this study, the continuous constitutive model was compiled in a developed subroutine VUHARD into the FE software ABAQUS 6.14 to achieve an accurate description of the welded tube mechanical properties in simulating tube free hydro-bulging. The mechanical properties of the welded tube were determined by matching the flow stresses to the welded tube node coordinates. The subroutine was programmed in the FORTRAN language [47]. The flowchart of the VUHARD subroutine is shown in Figure 12.

### 4.2. Evaluating the Continuous Constitutive Model

#### 4.2.1. The Mises Stress Distribution during Free Hydro-Bulging of the Welded Tube

**Figure 13:**
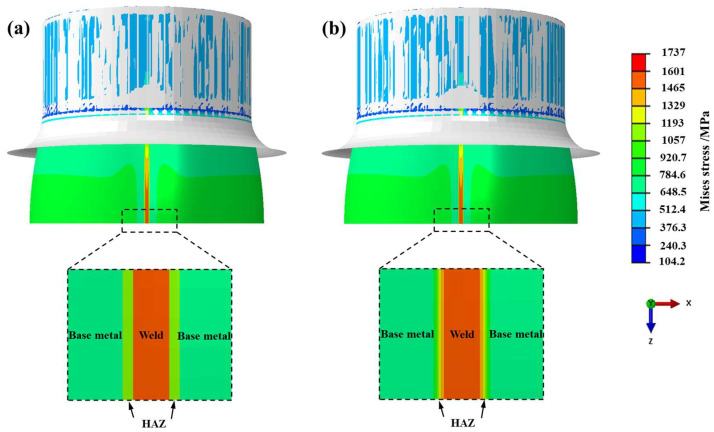
The Mises stress contour of BR1500HS welded tube in free hydro-bulging: (**a**) discrete constitutive model and (**b**) continuous constitutive model.

The Mises stress contour during free hydro-bulging of BR1500HS welded tube is shown in Figure 13. Figure 13a shows the Mises stress contour for an inhomogeneous welded tube with the application of the discrete constitutive model. It was shown that under the bulging pressure, the base metal zone deformed before the weld zone owing to the lower yield strength than that of the weld. Thus, the Mises stress in the base metal zone on both sides of the weld zone of the welded tube was lower than the weld zone. There is a sharp change in the Mises stress of the welded tube at the material interface and do not conform to the material’s continuous characteristics in practice. Figure 13b shows the Mises stress contour for an inhomogeneous welded tube with the application of the continuous constitutive model. It was demonstrated that the welded tube realized a continuous change in the Mises stress at the material interface, and the sharp change in the stress was eliminated.

**Figure 14:**
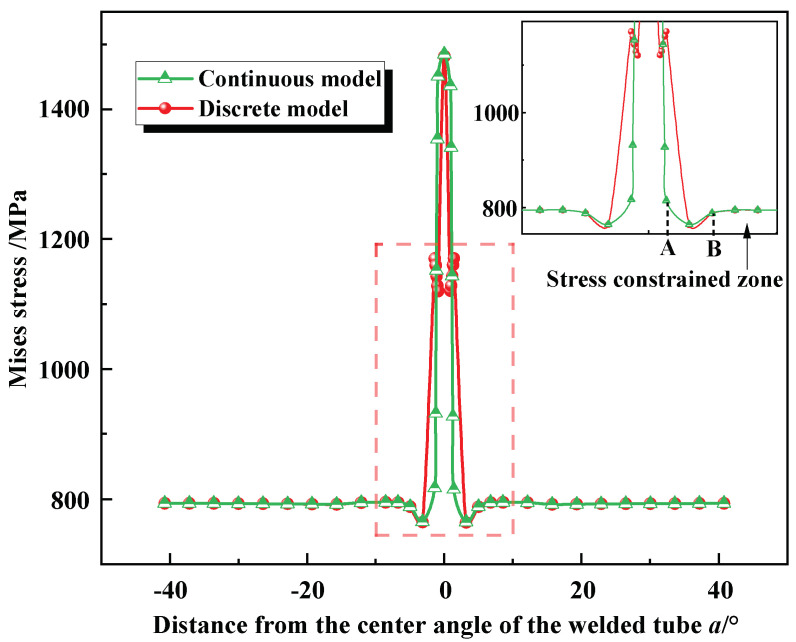
The Mises stress along the center angle of the welded tube.

Figure 14 shows the Mises stress distribution of the circumferential in the middle of the BR1500HS welded tube during free hydro-bulging simulation, including the results of both continuous and discrete models. It was obvious from Figure 14 that both models can reflect the welded tube’s mechanical properties during free hydro-bulging. However, the Mises stress curves of the discrete constitutive model had obvious sharp change characteristics, which would affect the accuracy of the simulation results. In contrast, the Mises stress of the continuous constitutive model varied continuously along the center angle of the welded tube, eliminating sharp change in material properties at the interface in the narrow welded joint zone. In addition, lower Mises stress exists between the parent metal zone and HAZ interface (point A) and the parent metal zone (point B). Therefore, the inhomogeneous mechanical properties of the welded tube have significant stress constraints on the base metal zone close to the weld during the bulging process (magnified area in the dotted box of Figure 14). That is, under the influence of the higher yield strength in the weld zone and the HAZ, larger plastic deformation up to rupture occurs in the higher stress constrained zone.

#### 4.2.2. The Equivalent Strain Distribution during Free Hydro-Bulging of the Welded Tube

**Figure 15:**
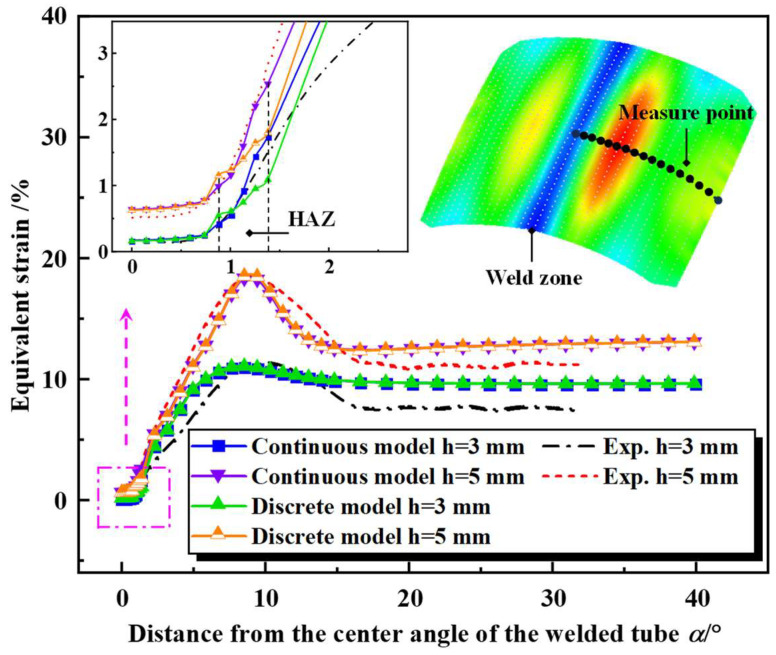
Comparison of equivalent strain between the experimental and simulation.

To evaluate the established continuous constitutive model, the equivalent strain during the bulging process of the welded tube was considered for comparison. The equivalent strain along the weld to the base metal for different bulging heights (3 mm and 5 mm) was extracted from simulation and experiment based on specific paths, as demonstrated in Figure 15. As seen in Figure 15, the equivalent strain predicted by the two models was well consistent in trend with the experimentally measured. In addition, the two models successfully predicted the maximum equivalent strain for different bulging height (h) conditions. However, the continuous constitutive model exhibited higher prediction accuracy in the HAZ than the discrete constitutive model. For instance, the average error in the equivalent strain predicted by the discrete constitutive model was 19.29% and 22.08% at the bulging height of 3 mm and 5 mm, respectively; in contrast, the average error of the equivalent strain predicted by the continuous constitutive model was 10.43% and 7.57%, respectively. Therefore, the deformation response of welded tubes during free hydro-bulging can be accurately described using the established continuous constitutive model.

Although the equivalent strain predicted by the two models agrees with the experimental trend, there was a significant prediction error when the welded tube center angle was larger than 15°. The reason for the above differences is probably attributed to the wall thickness differences between the experiment and FE simulation. In the practical tube manufacturing process, the wall thickness distribution of the welded tube used in the experiment was non-uniform. While the welded tube was defined as a uniform wall thickness distribution in the simulation.

#### 4.2.3. The Wall Thinning Rate Distribution during Free Hydro-Bulging of the Welded Tube

To further verify the accuracy of the continuous constitutive model that has been established, the wall thinning rate during the bulging process of the welded tube was considered for comparison. The wall thickness change in the tube can be expressed by Equation (9). Where *t*_0_ and *t* are the wall thickness of the tube before and after bulging, respectively.

(9) $$\Delta t=(t_{0}-t)/t_{0}\times 100\%$$

The instantaneous wall thickness *t* during the tube free hydro-bulging can be determined by Equation (10) according to the principle of constant volume. Where *ε*_1_ and *ε*_2_ are the hoop and axial strains of the tube, respectively. The hoop and axial strains during the tube bulging experiment can be acquired by the DIC strain measurement system in real time. Combining Equations (9) and (10), the wall thinning comparative results for experimental and simulated rates can be obtained.

(10) $$\varepsilon_{1}+\varepsilon_{2}+ln(\frac{t}{t_{0}})=0$$

**Figure 16:**
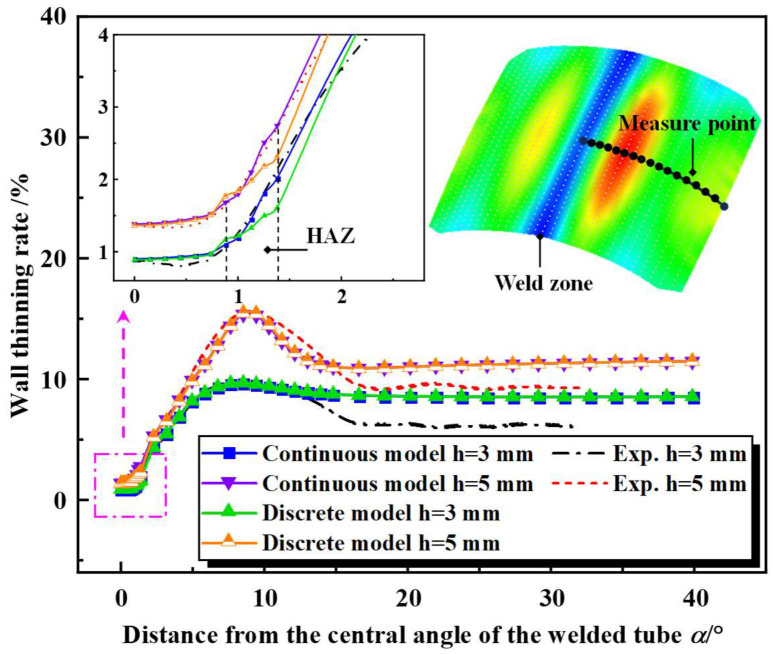
Comparison of the wall thinning rate between the experimental and simulation.

The wall thinning rate along the weld to the base metal was extracted based on the specific path shown in Figure 16 for simulation and experiment at different bulging heights (3 mm and 5 mm). As can be seen in Figure 16, the predicted wall thinning rate of the two models had the same trend as the experimentally measured when the center angle of the welded tube was less than 15°. In addition, the two models successfully predicted the maximum wall thinning rate and its location for different bulging height conditions. The weld and the HAZ show less deformation than the base metal because they have higher strength than the base metal. Therefore, the weld and the HAZ had a relatively low wall thinning rates, while the maximum wall thinning rate was located in the base metal zone near the weld which was subjected to a larger stress constraint (8.54° distance from the weld center). The continuous constitutive model had higher prediction accuracy in the HAZ than discrete constitutive model for the wall thinning rate. For instance, the average error in the wall thinning rate predicted by the discrete constitutive model was 13.64% and 8.02% at the bulging height of 3 mm and 5 mm, respectively; in contrast, the average error of the wall thinning rate predicted by the continuous constitutive model was 3.97% and 1.97%, respectively. Therefore, the free hydro-bulging deformation behavior of welded tubes can be accurately predicted using the established continuous constitutive model.

## 5. Conclusions

In this paper, an accurate continuous constitutive model of welded tubes was developed to improve the accuracy in simulating the complex plastic forming process of welded tubes and its application in practice in hydroforming industries. The main conclusions are as follows:

(1) In this study, the isothermal treatment method was proposed to characterize the microstructure of welded tube joints with a sharp property change. The relationship between the mechanical properties and the center angle of the welded tube under different conditions was obtained. When the isothermal transformation time increased from 30 s to 90 s, the flow stress decreased significantly. The tensile strength of the tensile specimen decreased from 1452 MPa to 1095 MPa, and the Vickers hardness decreased from 410.7 HV0.2 to 286.3 HV0.2.
(2) The boron steel BR1500HS laser-welded tube continuous constitutive model was developed. The results demonstrated that the established continuous constitutive model could reveal the change in the flow stress in the welded tube joint zone more accurately and continuously.
(3) The FE simulation based on the established welded tube continuous constitutive model and the experiment of tube free hydro-bulging achieved good agreement. The complex deformation process particularly in the inhomogeneous zone (welded joint zone) of the welded tubes under a biaxial stress state was accurately simulated.
(4) The mechanical properties obtained by the isothermal treatment method in combination with the microstructure analysis were an effective way to determine the continuous constitutive model of welded tubes. This also provides an effective way to further study the plastic deformation behavior of welded tubes.

## Figures and Tables

**Table 1:** Chemical composition of the boron steel BR1500HS sheet (wt %).

C	Si	Mn	P	S	Cr	Ti	B	Al	Fe
0.2	0.4	1.2	0.02	0.015	0.25	0.04	0.002	0.04	Bal.

**Table 2:** Phase volume fraction of tested specimens and holding times for ferrite formation.

Specimens	Ferrite (vol%)	Martensite (vol%)	Ferrite Formation Time *t*_f_ (s)	Contact Pressure (MPa)	Die Quenching time (s)
M	1.5	98.5	0	15	15
FM-1	17.4	82.6	30	15	15
FM-2	43.6	56.4	60	15	15
FM-3	60.1	39.9	90	15	15

**Table 3:** Mechanical property parameters in various zones of the welded tube.

BR1500HS Welded Tube	Yield Strength MPa	Tensile Strength MPa	Strength Coefficient *K*/MPa	Hardening Index *n*	*COD(R^2^)*
Weld zone	1205	1654	4273.656	0.253	0.96735
Sub-critical HAZ-1	1024	1452	3263.788	0.222	0.95667
Sub-critical HAZ-2	861	1339	2741.040	0.202	0.95430
Sub-critical HAZ-3	657	1095	1977.789	0.180	0.96808
Base metal zone	588	696	987.972	0.107	0.97104

## Data Availability

Data are contained within the article.
